# The attitude and behaviors of the different spheres of the community of the United Arab Emirates toward the clinical utility and bioethics of secondary genetic findings: a cross-sectional study

**DOI:** 10.1186/s40246-023-00548-7

**Published:** 2023-11-06

**Authors:** Azhar T. Rahma, Aminu S. Abdullahi, Giulia Graziano, Iffat Elbarazi

**Affiliations:** 1 Institute of Public Health, College of Medicine and Health Sciences, United Arab Emirates University, Al Ain, 15551 UAE; 2 Sapienza Università di Roma, Rome, Italy

**Keywords:** Healthcare providers, Academia, Diabetes, Blindness, Gender

## Abstract

**Introduction:**

Genome sequencing has utility, however, it may reveal secondary findings. While Western bioethicists have been occupied with managing secondary findings, specialists’ attention in the Arabic countries has not yet been captured. We aim to explore the attitude of the United Arab Emirates (UAE) population toward secondary findings.

**Method:**

We conducted a cross-sectional study between July and December 2022. The validated questionnaire was administered in English. The questionnaire consists of six sections addressing topics such as demographics, reactions to hypothetical genetic test results, disclosure of mutations to family members, willingness to seek genetic testing, and attitudes toward consanguinity. Chi-squared and Fisher’s exact tests were used to investigate associations between categorical variables.

**Results:**

We had 343 participants of which the majority were female (67%). About four-fifths (82%) were willing to know the secondary findings, whether the condition has treatment or not. The most likely action to take among the participants was to know the secondary findings, so they can make life choices (61%).

**Conclusion:**

These results can construct the framework of the bioethics of disclosing secondary findings in the Arab regions.

**Supplementary Information:**

The online version contains supplementary material available at 10.1186/s40246-023-00548-7.

## Introduction

Exome/genome sequencing is becoming widely available, offering several advantages. Whole-exome sequencing (WES) and whole-genome sequencing (WGS) provide a valuable opportunity to learn about rare developmental diseases (“primary” findings) [1]. Despite its utility and benefits, WES and WGS may also reveal “secondary” (or “incidental,” “additional”) findings (SFs), which means genetic information not concerning the patient’s current situation, including pathogenic variants. Genome sequencing may lead to the detection of numerous variants, which may provide information on the late onset of diseases or the risk of transmitting them. Consequently, these data may be useful to patients with rare diseases and their families [2].

Nowadays, the main issue concerns how secondary findings (SFs) should be investigated and communicated to patients [3]. According to Christenhusz et al. (2013), various factors should be considered when deciding about disclosing SFs in genetic contexts. Practical and technical factors involve the clinical utility of the finding (seriousness, urgency, treatability, impact on quality of life, probability, and disease context); scientific factors (whether the findings have been replicated, their robustness and quality, intentionality, and extent and complexity), and communication factors (who should disclose and to whom, communication capacity of the team, comprehension capacity of the recipients, family dynamics; possible/necessary consultation of colleagues/IRB) [4].

Ethical factors focus on maximizing benefits and minimizing harm. Disclosing SFs may indeed cause potential risks to privacy and confidentiality, as well as discrimination and psychological harm (fear, anxiety, confusion). Respect for autonomy and choice should be valued through the process of informed decision making. However, while some authors recognize the right not to know, others highlight patients’ need to know in specific life-threatening or life-changing cases [4–6]. Hiromoto et al. (2023) suggested some solutions to the difficulties aforementioned, such as improving genetic literacy, developing a consulting system with experts in the relevant disease, formulating guidelines, providing genetic counseling, and providing insurance coverage for medical care to unaffected carriers [7].

Even though, as already highlighted, benefits can be derived from SFs in terms of prevention, it is necessary to also consider patients’ points of view. In general, medical professionals are favorable to the disclosure of clinically relevant SFs [4]. Previous studies report participants’ desire to receive results and disclosure of genomic SFs, especially when they need to take preventive or therapeutic actions, to make an informed decision regarding different aspects of their life (career, reproduction, familial support, leisure), and to contribute to research advancement [3]. However, in some cases, patients are not interested in receiving genomic SFs [1].

According to Hiromoto et al. (2023), anticipatory guidance and confirmation of willingness for SFs disclosure from the client should be mandatory in order not to cause un-due anxiety. Moreover, after SFs disclosure, the client’s emotions need to be supported through an empathic and professional relationship [7]. These decisions depend on several factors including the type, conclusiveness, and nature of the results [4], as well as participants’ values, disease experiences and perceptions, priorities in life, and self-perceived ability to endure negative psychological effects [3]. Another likely action to highlight is the importance of confirming the patient's willingness for secondary findings disclosure and providing emotional support after disclosure to address potential anxiety and emotional reactions [8].

From a legal point of view, Jiang (2022) provides an examination of SF recommendations of the leading countries in genomics research and practice: the United States (US), the United Kingdom (UK), Canada, Australia, Germany, Denmark, and the European Un-ion (EU). For purposes of brevity, only US and EU guidelines are reported [9].

Secondary findings include future clinical risks, including privacy and confidentiality concerns, psychological stress, disruption of family dynamics, ethical dilemma related to disclosure, need for medical decision making, reproductive choices influenced by genetic information, long-term healthcare management, increased use of health resources as well as possible ethical and emotional challenges [10, 11].

In 2013, the American College for Medical Genetics and Genomics (ACMG) recommended that “laboratories and clinics utilizing whole-genome sequencing/whole-exome sequencing should have clear policies in place related to disclosure of SFs. Patients should be informed of those policies and the types of SFs that will be reported back to them and under what circumstances. Patients should be given the option of not receiving certain SFs.” Despite this, there is still no universally accepted reporting method [4, 12]. In 2014, the ACMG Board of Directors created the ACMG Secondary Findings Maintenance Working Group to define and update the SF gene list. Nominated genes should be medically actionable, have apparent phenotype associated with disease-causing variants, have severe medical implications for at least one of the phenotypes associated with the gene, and be associated with a highly penetrant phenotype [9].

In 2017 and again in 2021, ACMG updated the guidelines and extended the list of gene-disease pairs to additional SFs [9, 13, 14]. Meanwhile, in 2013, the European Society of Human Genetics (ESHG) guidelines recommended avoiding SFs that are not interpretable or medically actionable. On the contrary, if an SF indicates a severe health problem and is medically actionable, it should be reported to the patient, overriding the patient’s desire not to know [9].

While Western bioethicists have been occupied with the issue of managing incidental findings, specialists’ attention in the Arabic countries has not yet been captured and it is still in its infancy [15]. However, in Middle East countries, 60–70% of all marriages occur between first cousins, leading to uniquely common genetic disorders compared to Western countries [16]. Despite this, in the Arab countries the issue of genetic testing has been addressed only partially, and few studies have examined the attitude of patients or communities toward SFs [17]. As these nations, especially in the Gulf region, transform their healthcare systems toward personalized medicine, they need to address certain major ethical issues [15].

Pharmacogenomic testing is a type of genetic testing that analyzes an individual's genetic makeup to determine how they may respond to certain medications [18]. Pharmacogenomic counseling involves interpreting the results of this testing and providing recommendations to healthcare providers regarding the selection and dosing of medications for individual patients [19]. In the context of preemptive WGS, pharmacogenomic counseling can be used to provide preemptive guidance regarding medication selection and dosing based on an individual's genetic makeup. However, the use of preemptive WGS and pharmacogenomic counseling raises ethical considerations regarding the reporting of incidental findings and the potential for psychological harm caused by discovering unexpected genetic information. Healthcare providers must balance the potential benefits of preemptive genetic testing with the potential risks and must ensure that patients are fully informed about the potential outcomes of such testing [20].

Converging on the attitude of healthcare providers toward secondary findings, a quantitative study conducted on Pediatric Experts in Chicago revealed that above 80% thought that patients and parents should have the right to decline the disclosure of secondary findings [21]. Another qualitative focus group study disclosed that internal medicine and pediatric geneticists are relatively not competent in deciphering the SF results of pharmacogenomics testing devoid of the assistance of scientific resources [22].

Breadth of literature tackled the attitude and involvement of pharmacists in genetic testing and voiced their concerns regarding primary genetic testing [23–29] and even a review by Mills and Haga (2013) proposed a collaboration among genetic counselors and pharmacists to aid in the thorough counseling of genetic test results [30]. However, paucity of studies addressed the attitude of pharmacists toward secondary findings. Keeping in mind that their attitude may impact their practice and patient’s counseling sessions. Availability of direct-to-consumer kits in the community pharmacies will eventually expose pharmacists to scenarios where they have to interpret the results of such tests without the support of genetic counselors. We aim to explore the attitude of different spheres of the multiethnic community in the United Arab Emirates toward secondary findings in terms of their clinical utility and addressing the descriptive empirical aspect of bioethics.

## Materials and methods

We performed a cross-sectional study using a validated and piloted questionnaire (Additional file 1). The pursued sample included different spheres of the community (pharmacists, pharmacy technicians, physicians, nurses, radiologists, laboratory personnel, public health, academia including faculty and undergraduate and postgraduate students, and other non-medical professions) in the United Arab Emirates (UAE). We utilized convenient sampling techniques as well as snowball sampling where existing participants recruit future subjects from among their acquaintances that meet our inclusion criteria. The survey was created using SurveyMonkey software and was administered electronically between July and December 2022. The questionnaire was constructed based on the literature to explore the attitudes and behavior of community toward secondary findings. We piloted the questionnaire among 10 participants and sought out expert opinion for comments. Readability scores are: Flesch Reading Ease test for the survey was 63.2; moreover, the Flesch–Kincaid grade level test was 7.7. Our participant recruitment strategy encompassed various techniques: email, WhatsApp, LinkedIn, Facebook, and different social media platforms. The technique commenced with initial outreach through email, wherein survey participants were provided with an information sheet and a link to the survey. Once the participants opened the link of the survey, the participants were asked “Do you agree to participate in this survey?” and only those who said “Yes, I will take this survey” were able to access the questionnaire.

Moreover, in the information sheet it was stated that “you may withdraw at any time from the study. Please note that all of the information that will be collected through this questionnaire will be treated with strict confidentiality. We will not ask you for any personal information that may identify you. All of our data will only be accessed for data analysis purposes only.” Furthermore, to ensure transparency and fine practices, we adhered to the Checklist for Reporting Results of Internet E-Surveys (CHERRIES) [31].

The questionnaire was administered in English, and it allotted six sections:

1. *Demographic* gender, social status, having children, area of residence, age, education level, occupation, monthly salary, if medically insured, if conducted a genetic test, and if been advised to conduct a genetic test.
2. *Scenario 1* let us imagine the following scenario, you conducted a DNA test to check if you are a carrier of a genetic mutation that predicts diabetes. The report came back with a secondary finding that you are a carrier of the genetic mutation for another disease. Would you like to know the result of the other diseases that you did not test for (secondary findings)? The options are A-Yes, I want to know even if this other disease has treatment or not. B-Yes, I want to know ONLY if this other disease has treatment. C-No, I do not want to know, I did not test for this new disease. D-I do not know.
3. *Scenario 2* let us imagine, that the doctor is explaining the secondary findings to you, and you are carrying a mutation that predicts that you may be blind in the future, what you will do? The actions are A-Ask your doctor NOT to tell you the results. B-Ask your doctor to tell you the result, ONLY if there is TREATMENT NOW for blindness. C-Ask your doctor to tell you the result, ONLY if there is LIFESTYLE modification that you can do. D-Ask your doctor to tell you because you want to inform your CAREGIVERS, so they can act. E-Ask your doctor to tell you, so YOU can make life choices, like switching jobs, fixing your home, finding a driver etc. F-Ask your doctor NOT to document this information in your file, so you do not lose your current insurance. G-Ask your doctor NOT to document this information in your file, so you your employer does not know, and you do not lose your current job.
4. *Disclosure* Are you going to tell your siblings about this mutation, so they can do the test themselves? Are you going to tell your children about this mutation, so they can do the test themselves?
5. *Willing to seek genetic testing* If a member of your family had a genetic disease, are you willing to take a genetic test and seek genetic counseling to determine if you have that genetic condition or not?
6. *Attitude toward consanguinity* Do you think a person with a genetic disease or at risk for one can marry his or her cousin?

We calculated the sample size using the WHO sample size calculator (sample-size-calculator.xls (live.com), and our estimated sample size was 384. The variables for the sample size calculations are: 95% confidence level, margin of error (MOE) is 0.05, baseline levels of the indicators are 0.5 and design effect is 1. This study had been approved by the Social Science Research Ethics Committee of United Arab Emirates University (UAEU) ERS_2017_5671.

Data were analyzed using descriptive and inferential statistics. Categorical variables were reported using frequencies and percentages while the continuous variable age was summarized using median and interquartile range. Variables were cross-tabulated to identify relationships. Observed relationships between variables were inferentially explored using the Chi-squared test or Fisher’s exact test as appropriate, and logistic regression analysis with a 5% level significance. All data analyses were performed using R software version 4.1.2 [32].

## Results

A total of 343 people participated in the study, most of whom were female (67%). The median age of respondents was 35 years old (IQR = 28, 42). Slightly more than half were married (56%) and had no children (51%). Mostly, the participants had either a postgraduate level of education (55%) or a bachelor’s degree (40%), as only 5.5% had a secondary or diploma level of education (Table 1). About four in five participants (82%) had a monthly salary of more than 10,000 AED. The majority (71%) were employed with about half (51%) having a health-related job.

**Table 1:** Characteristics of the study population (*N* = 343)

Characteristic	*N* (%)
*Age, median (IQR)*	35 (28, 42)
*Gender*	
Female	231 (67%)
Male	112 (33%)
*Nationality*	
UAE	67 (20%)
Expats	274 (80%)
*Marital status*	
Single	141 (41%)
Married	191 (56%)
Divorced/separated/widowed	11 (3.2%)
*Highest education*	
High school or diploma	19 (5.5%)
Bachelor’s degree	138 (40%)
Master’s degree	112 (33%)
PhD	74 (22%)
*Employment status*	
Unemployed	99 (29%)
Employed	243 (71%)
*Occupation*	
Health-related	121 (51%)
Other professions	106 (45%)
Students	10 (4.2%)
*Monthly salary (AED)*	
< 3000	12 (5.2%)
3000–10,000	62 (27%)
11,000–20,000	86 (37%)
> 20,000	71 (31%)
*Have children (yes)*	176 (51%)

Some variables may not add to 343 due to missing data

*AED* UAE Dirham

Overall, only 12% of the participants were advised by their doctors to take a genetic test, as 10% reported never having a genetic test. Across professions, the majority of the participants reported neither being advised by a doctor to take a genetic test (84–100%) nor they had ever taken a genetic test (89–100%). Those in healthcare professions were more likely to have been advised by their doctors to take a genetic test (13% versus 11%) or to have taken a genetic test (11% versus 9%) than those working in other professions. Moreover, none of the student participants reported either being advised to take a genetic test or taking a genetic test.

Being advised by a doctor to take a genetic test was found to be significantly associated with having a genetic test even after adjusting for possible confounding effect of age and gender (AOR = 5.87, 95% CI (Table 2).

**Table 2:** Logistic regression analysis for factors associated with taking genetic test

Factor	Crude OR	95% CI	*p* value	Adusted OR	95% CI	*p* value
*Age*	0.98	0.94, 1.02	0.3	0.99	0.95, 1.03	0.7
*Gender*						
Female	1	–		1	–	
Male	1.5	0.70, 3.16	0.3	1.53	0.68, 3.38	0.3
*Advised to take a genetic test*						
No	1	–		1	–	
Yes	6.24	2.67, 14.3	** < 0.001**	5.87	2.46, 13.8	** < 0.001**

Bold values indicate statistical significance

**Fig. 1:**
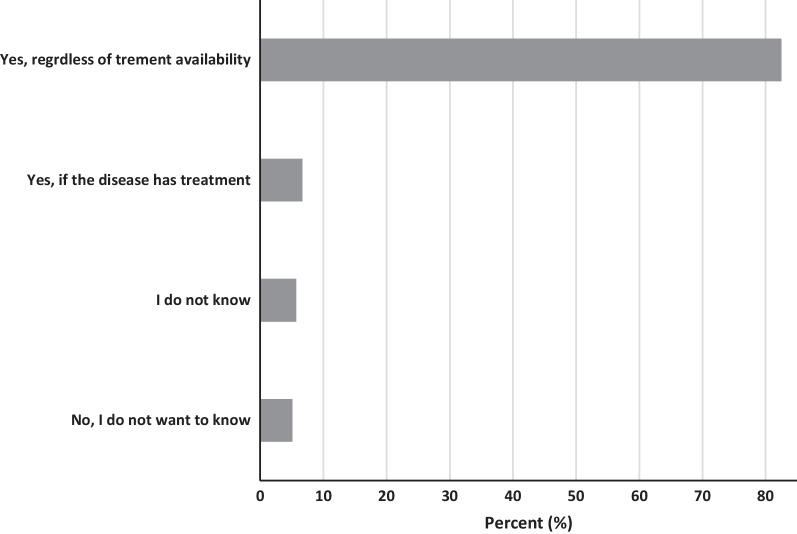
Willingness to know secondary results of genetic test

Willingness to know about one’s secondary findings of a genetic test was generally high among the participants with more than four-fifths (83%) expressing their willingness to know the secondary findings, irrespective of whether the diagnosed secondary condition has a treatment or not (Fig. 1). An additional 7% were also willing to know the secondary findings but only if the conditions had a treatment. The rest (11%) were either unsure (6%) or did not want to know (5%).

Upon a secondary genetic test finding that predicts future blindness, the most likely action to take among the participants was to “ask their doctor to tell them, so they can make life choices, like switching jobs, fixing their home, finding a driver, etc.” (61%), followed by “ask their doctor to tell them, because they would want to inform their caregivers, so they could take action” (39%), “ask their doctor to tell them, only if there is lifestyle modification that they can do” (26%), “ask their doctor not to document this information in their file, so their employer does not know and they do not lose their current job” (16%), “ask their doctor to tell them, only if there is treatment now for the blindness” (14%), “ask their doctor not to document this information in their file, so they do not lose their current insurance” (12%), and “ask their doctor not to tell them the results” (8%).

Moreover, those with a bachelor’s degree as highest level of education (33%) were significantly (*P* = 0.038) more likely to “ask their doctor to tell them, only if there is lifestyle modification that they can do” than those with other levels of education (14–29%); males were significantly more likely to “ask their doctor not to document this information in their file, so their employer does not know and they do not lose their current job” than females (49% versus 34%, *P* = 0.009); those who were unemployed were significantly more likely to “ask their doctor to tell them, only if there is treatment now for the blindness” than the employed (70% versus 58%, *P* = 0.045); non-Emiratis were significantly more likely to “ask their doctor not to document this information in their file, so they do not lose their current insurance,” to “ask their doctor not to tell them the results” compared to the Emiratis (13% versus 3%, *P* = 0.027; and 19% versus 5%, *P* = 0.010 respectively); finally, students were significantly more likely to “ask their doctor not to tell them the results” compared to those in other occupation categories (56% versus 11–16%, *P* = 0.006) (Table 3).

**Table 3:** Actions toward secondary findings that predict future blindness by demographic characteristics

Characteristic	Action 1		Action 2		Action 3		Action 4		Action 5		Action 6		Action 7	
	Yes	*P*	Yes	*P*	Yes	*P*	Yes	*P*	Yes	*P*	Yes	*P*	Yes	*P*
	*n* (%)	value	*n* (%)	value	*n* (%)	value	*n* (%)	value	*n* (%)	value	*n* (%)	value	*n* (%)	value
Gender		0.348		0.621		0.417		**0.009**		0.725		0.597		0.35
Female	15 (7.0%)		28 (13%)		59 (28%)		73 (34%)		132 (62%)		26 (12%)		37 (17%)	
Male	10 (10%)		15 (15%)		23 (23%)		49 (49%)		59 (60%)		10 (10%)		13 (13%)	
Employment		0.167		0.717		0.236		0.946		**0.045**		0.438		0.479
Yes	21 (9.3%)		32 (14%)		55 (24%)		87 (39%)		130 (58%)		24 (11%)		34 (15%)	
No	4 (4.6%)		11 (13%)		27 (31%)		34 (39%)		61 (70%)		12 (14%)		16 (18%)	
Occupation		0.774		0.885		0.1		0.424		0.274		0.118		**0.006**
Health-related	11 (9.6%)		18 (16%)		35 (31%)		40 (35%)		66 (58%)		11 (9.6%)		13 (11%)	
Other professions	8 (8.0%)		13 (13%)		19 (19%)		43 (43%)		61 (61%)		10 (10%)		16 (16%)	
Students	1 (11%)		1 (11%)		1 (11%)		4 (44%)		3 (33%)		3 (33%)		5 (56%)	
Marital status		0.134		0.65		0.157		0.306		0.427		0.232		0.32
Single	6 (4.8%)		20 (16%)		39 (31%)		42 (34%)		81 (65%)		14 (11%)		22 (18%)	
Married	19 (11%)		22 (12%)		42 (24%)		75 (42%)		103 (58%)		19 (11%)		25 (14%)	
Divorced/separated/widowed	0 (0%)		1 (9.1%)		1 (9.1%)		5 (45%)		7 (64%)		3 (27%)		3 (27%)	
Highest education		0.228		0.782		**0.038**		0.967		0.078		0.107		0.509
Secondary or Diploma	3 (18%)		2 (12%)		5 (29%)		6 (35%)		9 (53%)		2 (12%)		4 (24%)	
Bachelor	12 (9.7%)		19 (15%)		41 (33%)		47 (38%)		69 (56%)		8 (6.5%)		16 (13%)	
Masters	7 (6.8%)		15 (15%)		26 (25%)		41 (40%)		62 (60%)		15 (15%)		17 (17%)	
PhD	3 (4.3%)		7 (10%)		10 (14%)		28 (41%)		51 (74%)		11 (16%)		13 (19%)	
Nationality		0.287		0.471		0.076		0.057		0.455		**0.027**		**0.010**
Emiratis	7 (12%)		10 (17%)		21 (35%)		17 (28%)		34 (57%)		2 (3.3%)		3 (5.0%)	
Non-Emiratis	18 (7.1%)		33 (13%)		60 (24%)		105 (42%)		156 (62%)		34 (13%)		47 (19%)	
Insurance		0.706		> 0.999		0.787		0.342		0.249		0.187		0.094
Yes	24 (8.4%)		3 (12%)		76 (26%)		110 (38%)		173 (60%)		31 (11%)		43 (15%)	
No	1 (4.0%)		40 (14%)		6 (24%)		12 (48%)		18 (72%)		5 (20%)		7 (28%)	

Action 1: Ask your doctor NOT to tell you the results

Action 2: Ask your doctor to tell you the result, ONLY if there is TREATMENT NOW for the blindness

Action 3: Ask your doctor to tell you the result, ONLY if there is LIFESTYLE modification that you can do

Action 4: Ask your doctor to tell you, because you want to inform your CAREGIVERS, so they can take actions

Action 5: Ask your doctor to tell you, so YOU can make life choices, like switching jobs, fixing your home, finding a driver

Action 6: Ask your doctor NOT to document this information in your file, so you do not lose your current insurance

Action 7: Ask your doctor NOT to document this information in your file, so you your employer does not know

Bold values indicate statistical significance

Moreover, the majority of the participants would tell their siblings (77%) and/or their children (56%) about the secondary genetic test results that predict future blindness. Furthermore, married (13%) and widowed/divorced/separated (27%) participants were significantly (*P* < 0.001) more likely to tell their children about the blindness-related secondary findings if the children were up to 21 years old compared to single participants (Table 4).

**Table 4:** Willingness to tell siblings and children about secondary findings that predict future blindness by demographic characteristics (*N* = 312)

Characteristic	Tell siblings			Tell children		
	Yes, *n* = 241	*P* value	Yes, *n* = 176	*P* value	Yes, if ≥ 21 years, *n* = 29	*P* value
	*n* (%)		*n* (%)		*n* (%)	
Gender		0.384		0.460		0.563
Female	166 (79%)		116 (55%)		21 (10%)	
Male	75 (74%)		60 (59%)		8 (7.9%)	
Occupation		0.342		0.765		0.676
Health-related	87 (75%)		68 (59%)		14 (12%)	
Other professions	75 (77%)		6 (55%)		9 (9.3%)	
Students	5 (56%)		4 (44%)		9 (100%)	
Marital status		0.277		0.081		** < 0.001**
Single	99 (82%)		59 (49%)		3 (2.5%)	
Married	133 (74%)		111 (62%)		23 (13%)	
Divorced/separated/widowed	9 (82%)		6 (55%)		3 (27%)	
Highest education		0.868		0.386		0.807
Secondary or Diploma	14 (82%)		12 (71%)		2 (12%)	
Bachelor’s degree	98 (79%)		65 (52%)		11 (8.9%)	
Master’s degree	78 (76%)		57 (55%)		11 (11%)	
PhD	51 (75%)		42 (62%)		5 (7.4%)	
Nationality		0.86		0.703		0.796
Emiratis	46 (78%)		32 (54%)		5 (8.5%)	
Non-Emiratis	193 (77%)		143 (57%)		24 (9.6%)	
Insurance		0.212		0.531		> 0.999
Yes	220 (76%)		161 (56%)		27 (9.4%)	
No	56 (86%)		110 (41%)		2 (6.9%)	

Bold values indicate statistical significance

**Fig. 2:**
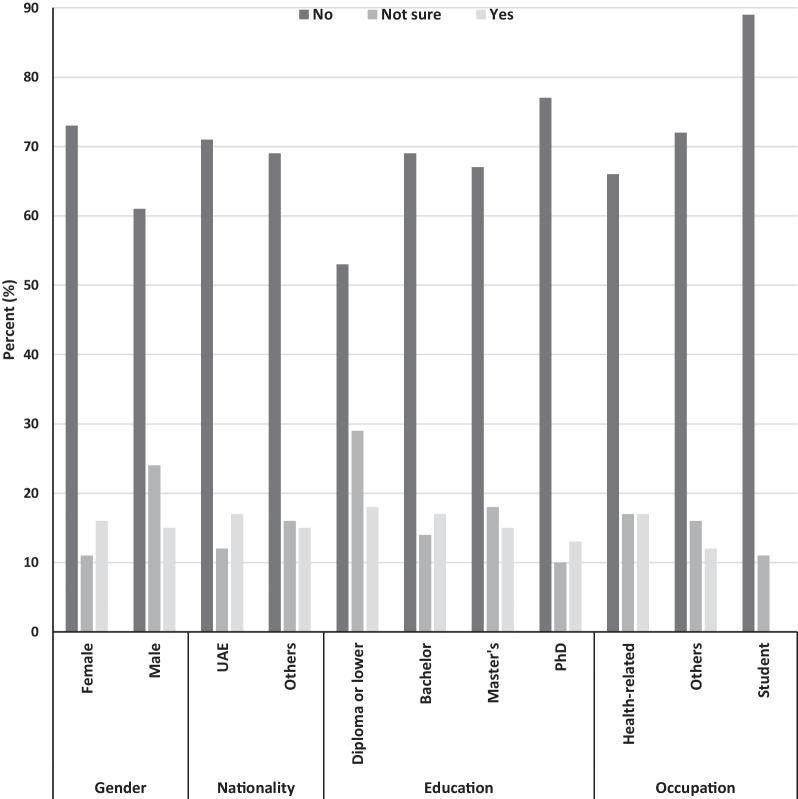
Can someone with a genetic disease marry their cousin?

When asked about whether one with a genetic disease could marry their cousins, the majority of the participants said: “no” (69%), others said, “they were not sure” (16%), while the rest said “yes” (15%). Figure 2 depicts the distribution of the responses to the question of whether a person with a genetic disease could marry their cousins by demographic characteristics.

## Discussion

The ethical dilemma of disclosing secondary findings to the person on one level and to his family (siblings and children) on another level is the core of this research. Hence, surveying the attitude of many spheres of the multiethnic community of UAE toward secondary findings in terms of clinical utility and bioethics is pivotal to stakeholders. About four-fifths (82%) of the community in UAE were willing to know the secondary findings, whether the condition has treatment or not. The most likely action to take among the participants was to know the secondary findings, so they can make life choices and adjustment (61%).

Results about pharmacists’ attitudes toward genetic testing are in consonance with the literature about the genetic literacy of pharmacists. As despite 17 percent of pharmacists in our cohort reporting being advised by a doctor to take a genetic test, only about six percent reported having taken the test. Works of literature from all over the globe have revealed that pharmacists have a poor level of knowledge about genetic tests as most of them have not studied this science at colleges and universities [27, 33–43]. This outcome has to be triangulated by the stakeholders in order to ensure effective implementation of precision medicine in their healthcare systems.

Uncovering the generally high attitude of our cohort within all job categories toward keenness to know about one’s secondary findings of genetic test, irrespective of whether the diagnosed secondary condition has treatment or not is in contrast with the European Society of Human Genetics (ESHG) guidelines [9]. Moreover, it has a profound repercussion in terms of the bioethics of disclosing secondary findings in the Arab regions and can gear the policy statements and guidelines in UAE, Gulf Corporation Council (GCC) and EMRO regions. The attitude of our cohort is in contrast with the general trend reported by Middleton et al. (2016) in their study of the mindsets of approximately 7000 health experts, genomic investigators, and public concerning secondary findings [44]. They reported an inverse relationship between the severity of the secondary findings and the positive attitude toward receiving these findings which was not the case in our cohort [44].

Upon a secondary genetic test finding that predicts future blindness, the most likely action to take among academics, pharmacists, and other healthcare workers was to ask the doctor to tell them the results, so that they could make life choices, like switching jobs, fixing their home, finding a driver as well as to inform their caregivers, so that they can take actions. Even though few studies explored these attitudes, however, a semi-structured interviews with 40 patients with advanced reported that most patients voiced interest in the possibility of discovering their secondary germline findings and viewed that as relevant to themselves: their families [45].

The resolution of our participants of knowing the secondary findings was tied to their intent to make life choices and adjustment for themselves or their caregivers. This attitude can be explained by the strong and extended family texture and their commitment to the preservation of life [46].

In our cohort, the majority of the participants would tell their siblings and children about the secondary genetic test results which is in line with previous study assessing attitude of the multiethnic population of the United Arab Emirates on genomic medicine and genetic testing [47]. Pharmacy professionals in our cohort have a reduced tendency of disclosure than their counterparts in health settings or those in academia. A case study from Turkey by Akpinar and Ersoy (2014) reported that 26% of physicians and 49% of patients judged that genetic results are in fact ownership of the entire family [48]. No studies addressed the disclosure attitude of pharmacists, and our study can encourage researchers to dwell into the reasons justifying their attitudes. Moreover, studies have shown that the presence of minors, the duties, and responsibilities of healthcare professionals, and the justice system have an impact on the process of decision making [4].

Gender had been observed to influence the attitude of our cohort, as females were more inclined to disclose their SFs with their siblings and with their children when they are above 21 years old. This attitude had been spotted in the literature, under the view of feminist ethics, as it is in women’s nature and instincts to care for and protect others [49].

About a quarter of the pharmacists in our sample contemplate that someone with a genetic disease can marry their cousin, which is a higher percentage in comparison with academia, but lower than other healthcare providers and non-healthcare providers. This stance can be explained by the effect of culture as illustrated in our proposed genomic literacy framework for pharmacists [50]. Worth noting that consanguinity is prevalent in the Arabic regions [51–54].

The strengths of this study are that it will add value to the literature by addressing the attitude of various sectors of the community toward secondary findings and by reaching a large sample of the population. Another strength is the scenarios presented tackle both treated and non-treated diseases which will guide the bioethicists in disclosing secondary findings. Our study is susceptible to sampling bias due to convenience sampling as well as not being able to calculate the response rate due to the snowball sampling technique. Additionally, the final sample cannot be pondered as representative of any particular group due to the biases introduced by the convenience and snowball sampling. Moreover, not asking about the disease status of the participants and financial obstacles are another limitation that may mask the explanation of their attitude. Though this research offers worthy data about the attitudes of the community toward the proposed scenarios, we cannot assume that this is how they will react to real-time situations and whether their attitude would be a mirror of their anticipated one.

Future researchers can exploit our findings to generate an in-depth qualitative research study and to study real-time scenarios. The future perspectives of the attitude of pharmacists, healthcare providers, and academia toward the clinical utility and bioethics of secondary genetic findings are likely to be shaped by ongoing research and developments in the field of genomics. As more evidence becomes available on the clinical utility of secondary genetic findings, there may be increasing acceptance of the value of preemptive genomic sequencing and the reporting of incidental findings. The positive attitude of our cohort toward disclosing secondary findings can shape the guidelines to be culturally accepted and desired by the patients and their families.

Pharmacists, as medication experts, are well positioned to provide pharmacogenomic counseling and help patients understand the implications of secondary genetic findings on medication therapy. As such, their attitudes toward the clinical utility and bioethics of secondary genetic findings will be critical in shaping how these findings are communicated to patients.

In addition, healthcare providers and academia will also play a crucial role in shaping the attitudes and perspectives around the clinical utility and bioethics of secondary genetic findings. Continued education and training in genomics will be important for healthcare providers to ensure they are equipped to effectively communicate genomic information to patients and make informed decisions about patient care.

Overall, the attitudes and perspectives of pharmacists, healthcare providers, and academia toward secondary genetic findings will continue to evolve as the field of genomics advances, and ongoing dialogue and collaboration will be necessary to ensure that ethical considerations and patient welfare remain at the forefront of clinical practice.

This study provides several policy implications for policy makers, particularly regarding the management of SF associated with genetic testing in multiethnic communities in the United Arab Emirates. The study results demonstrate a strong willingness of people to learn about SF, regardless of whether there are treatment options for the identified symptoms, which contradicts European guidelines and general trends in other regions doing. This high attitude toward SF has significant bioethical implications for disclosure practices in the Arab region and requires the development of policy statements and guidelines that incorporate this positive attitude. Furthermore, this study suggests that gender plays a role in disclosure attitudes, with women being more likely to share SF with family members, and in developing genetic testing-related policies such as the prevalence of consanguinity in the Arab region. Moreover, the proposed policy needs to factor the cultural context and SF disclosure. These findings highlight the need for comprehensive policies that respect cultural norms and individual preferences while ensuring effective implementation of precision medicine in health systems.

In establishing bioethical standards for handling secondary findings inside the Arab area, it is vital to uphold the concepts of justice, beneficence, non-maleficence, autonomy, and the utilitarian principle [55]. Justice necessitates growing pointers that recognize cultural diversity, ensuring equitable access to the advantages of genetic trying out, and guarding against discrimination rooted in genetic facts. Beneficence calls for prioritizing person well-being by using presenting complete genetic literacy and academic applications, empowering individuals with know-how, and facilitating access to genetic counseling to manual them via complicated moral selections. Non-maleficence compels policymakers to protect in opposition to potential harms associated with privacy and confidentiality dangers, strengthening records safety policies to protect individuals' genetic records [11, 56]. Finally, embracing autonomy manner respectes individuals' rights to make knowledgeable choices about secondary findings, while the utilitarian theory underscores the significance of guidelines that maximize typical nicely being and societal advantage. These concepts together tell guidelines that stability the moral dimensions of secondary findings, making sure they are each culturally touchy and almost beneficial to the Arab place's healthcare systems (Additional file 1).

## Conclusions

The present study provides valuable data which can guide stakeholders’ statements and policies toward SFs disclosure. This paper might help in producing specific national guidelines on SFs disclosure in the United Arab Emirates, and other Arab countries, that take in account the attitudes and stance of the community.

### Supplementary Information

**Additional file 1:** Supplementary.

## Data Availability

The data that support the findings of this study are available from the corresponding author, IE, upon reasonable request.
