# Supplementary material for: The attitude and behaviors of the different spheres of the community of the United Arab Emirates toward the clinical utility and bioethics of secondary genetic findings: a cross-sectional study
Source: Hum Genomics. 2023 Nov 6;17:98. doi: 10.1186/s40246-023-00548-7 (PMC10626730; doi:10.1186/s40246-023-00548-7)
Supplement: Supplementary file 1 — Additional file 1: Supplementary. [file 40246_2023_548_MOESM1_ESM.pdf]

Attitudes of the community to Secondary findings of genetic testing

You have been invited to take part in a study that aims to explore the attitude of the UAE community toward secondary findings of a genetic test. (Secondary findings are genetic test results that provide information about changes (variants) in a gene unrelated to the primary purpose of the testing)

Participation in this study will take 5 minutes. I appreciate your support and cooperation. You may withdraw at any time from the study.
Please note that all of the information that will be collected through this questionnaire will be treated with strict confidentiality. We will not ask you for any personal information that may identify you. All of our data will only be accessed for data analysis purposes only.
Thank you for participating in our survey. Your feedback is important. Kindly note there is no wrong or correct answer.

This study had been approved by the social science research ethics committee of United Arab Emirates University (UAEU) ERS_2017_5671.

Attitudes of the community to Secondary findings of genetic testing

* 1. Do you agree to participate in this survey?

- ◯ Yes, I will take this survey
- ◯ No, I do not want to take the survey

Attitudes of the community to Secondary findings of genetic testing

* 2. What is your gender?

- ◯ Male
- ◯ Female

* 3. What is your nationality?

- ◯ UAE
- ◯ Other (please specify)

[ ]

* 4. What is your social status?

- Single
- Married
- Separated
- Divorced
- Widowed

5. Do you have children?

- Yes
- No

* 6. Where do you live?

- Abu-Dhabi
- Dubai
- Sharjah
- Ajman
- Fujairah
- Umm Al Quain
- Ras Al Khaima
- Other (please specify)

* 7. How old are you?

* 8. What is your highest degree (if you are still studying choose the certificate you are studying for)?

- Primary school
- High school
- Diploma
- Bachelor
- Master
- PhD
- Not applicable
- Other (please specify)

* 9. Are you employed?

- Yes
- No

Attitudes of the community to Secondary findings of genetic testing

* 10. What is your occupation (if you are a student write that you are a student)?

11. What is your monthly salary?

- Less than 3,000 AED
- 3,000-10,000 AED
- 11,000 - 20,000 AED
- More than 20,000 AED
- Not applicable

* 12. Are you medically insured?

- Yes
- No

Attitudes of the community to Secondary findings of genetic testing

* 13. Have you conducted a DNA test (/Genetic testing)?

- Yes
- No
- Other (please specify)

* 14. let us imagine the following scenario, you conducted a DNA test to check if you are a carrier of a genetic mutation that predict diabetes. The report came back with a secondary finding that you are a carrier of the genetic mutation for another disease.

Would you like to know the result of the other diseases that you did not test for (secondary findings)?

- Yes, I want to know even if this other disease has treatment or not.
- Yes, I want to know ONLY if this other disease has treatment.
- No, I do not want to know. (I did not test for this new disease)
- I do not know
- Other (please specify)

* 15. let us imagine, that the doctor is explaining the secondary findings to you, and you are carrying a mutation that predicts that you may be blind in the future, what you will do? (you can choose more than one answer)

- [ ] Ask your doctor NOT to tell you the results.
- [ ] Ask your doctor to tell you the result, ONLY if there is TREATMENT NOW for the blindness.
- [ ] Ask your doctor to tell you the result, ONLY if there is LIFE-STYLE modification that you can do.
- [ ] Ask your doctor to tell you, because you want to inform your CAREGIVERS, so they can take actions.
- [ ] Ask your doctor to tell you, so YOU can make life choices, like switching jobs, fixing your home, finding a driver etc.
- [ ] Ask your doctor NOT to document this information in your file, so you do not lose your current insurance.
- [ ] Ask your doctor NOT to document this information in your file, so you your employer does not know and you do not lose your current job.
- [ ] Other (please specify)

* 16. Are you going to tell your siblings about this mutation, so they can do the test themselves?

- ( ) Yes
- ( ) No
- ( ) I do not have siblings
- ( ) I do not know
- ( ) Other (please specify)

* 17. Are you going to tell your children about this mutation, so they can do the test themselves?

- ( ) Yes
- ( ) No
- ( ) I do not have children
- ( ) I do not know
- ( ) I will tell them when they are above 21 years old
- ( ) Other (please specify)

* 18. Have you ever been advised by (a doctor, friend, relative, or medical representative) to do a genetic test?

- ( ) Yes
- ( ) No
- ( ) I do not remember

* 19. If a member of your family had a genetic disease, Are you willing to take a genetic test and seek genetic counseling to determine if you have that genetic condition or not?

- ◯ Yes
- ◯ No
- ◯ I am not sure

Attitudes of the community to Secondary findings of genetic testing

* 20. Do you think a person with a genetic disease or at risk for one should marry his or her cousin?

- ◯ Yes
- ◯ No
- ◯ I am not sure

Attitudes of the community to Secondary findings of genetic testing

21. Thank you for your cooperation. hope you be healthy always, please take care of yourself. Kindly contact Dr. Azhar Talal for questions, comments, and more information. 201280026@uaeu.ac.ae

[ ]
